# Effect of ultra-low temperature storage on the viability of pepper pollen and its implications for hybrid breeding

**DOI:** 10.3389/fpls.2025.1516016

**Published:** 2025-03-26

**Authors:** Kanghua Du, Da Zhang, Jixian Ma, Zhong Dan, Xianqin Wen, Weiwu Lv, Long Yang, Lingfeng Bao, Yirong Li, Guangping Chen, Jie Zhang, Wanfu Mu

**Affiliations:** ^1^ Institute of Tropical Eco-agriculture Science, Yunnan Academy of Agricultural Sciences, Yuanmou, Yunnan, China; ^2^ Yunnan Provincial Seed Management Station, Kunming, Yunnan, China; ^3^ College of Landscape and Horticulture, Yunnan Agricultural University, Kunming, Yunnan, China

**Keywords:** pepper, pollen, ultra-low temperature preservation, long-term storage, viability

## Abstract

Pollen plays a vital role in plant reproduction, acting as a carrier of male genetic material for fertilization and ensuring species propagation and the maintenance of biodiversity. Ultra-low temperature preservation of pollen provides a reliable method for long-term storage while preserving its viability, thereby facilitating crop breeding, genetic resource conservation, and ecological restoration. This study aimed to establish a method for long-term preservation of pepper pollen under ultra-low temperature conditions. Pollen was collected from unopened flowers during the peak flowering stage of pepper plants and subjected to sequential treatments including pollen dispersal, drying, dehydration (water content of pollen < 10%), sealing with inert gas (nitrogen), pre-cooling treatment, long-term preservation at −80°C, thawing treatment followed by artificial pollination. The results demonstrated that pepper pollen preserved for one year using our method maintained a pollination rate over 90%. Comparisons with fresh pollen (CK) indicated no significant differences in either the number or quality of hybrid seeds. This study establishes a theoretical and practical foundation for crop genetic breeding and germplasm conservation research, thereby facilitating the rapid advancement of hybrid breeding in pepper.

## Introduction

*Capsicum annuum* L. is one of the most popular spice and vegetable crops worldwide, originating from South and Central America. It comprises approximately 35 species within the *Capsicum* genus and is considered a significant economic crop globally (Basu et al., 2003; Barboza and De, 2005), and is widely cultivated worldwide. According to 2021 statistics, the global pepper cultivation area exceeded 8 million hectares, with a total production of 36.29 million tons of green peppers and 4.84 million tons of dried peppers. In China, the pepper cultivation area exceeded 3.2 million hectares, accounting for 40% of the global cultivated area (Saha et al., 2023). However, many cultivated pepper varieties are highly susceptible to a wide range of diseases and pests (DeWitt and Bosland, 1996; Mohan and Anilkumar, 2020). As a result, pepper improvement has become a primary focus of breeding programs worldwide.

Currently, the development of new crop varieties still primarily relies on hybrid breeding techniques. Pollen plays a crucial role in crop reproduction, acting as an essential carrier of male gametes for propagating the next generation in hybrid breeding (Ren et al., 2019; Broussard et al., 2023). However, pollen has a short lifespan (Ren et al., 2019; Dafni and Firmage, 2000), and asynchrony in flowering times between parental plants during hybridization often leads to challenges in successful pollination (Jia et al., 2022). This can lead to incomplete pollination of the female flowers or an excess of male pollen, resulting in pollen wastage and inefficiencies in the breeding process. Therefore, pollen preservation is one of the important means to solve these problems.

Cryopreservation is an advanced technique for the preservation of plant genetic resources (Langedijk et al., 2023). Over the past three to four decades, significant progress has been made in this field. Cryogenic storage facilities have been established globally with the aim of conserving species that are difficult to preserve using conventional methods (Li et al., 2019; Rajasekharan and Ganeshan, 2018). Ultra-low temperature preservation of pollen is a safe and practical method for maintaining long-term pollen viability, helping to overcome breeding challenges such as flowering asynchrony, insufficient pollen production, or pollen wastage (Saha et al., 2023). More importantly, it effectively prevents the loss of parental lines in pepper breeding programs. Some studies have demonstrated that pollen can be cryopreserved at -80°C, in liquid nitrogen (LN, -196°C), or in its vapor phase (-150°C to -180°C) (Zhang et al., 2006; Chen et al., 2013; Jia et al., 2022; Ma et al., 2024). With the development of research, scientists have successfully preserved pollen from various species of sexually reproducing plants, such as soybean (Jia et al., 2022), potato (Faltus et al., 2024), Luffa (Nagar et al., 2023), apple (Ćalić et al., 2021), rose (Marchant et al., 1992), camellia (Luna et al., 2001), plum and peony (Ren et al., 2022) and pepper (Karipidis and Douma, 2009; Mathad et al., 2013), under cryogenic conditions for long-term storage. We believe that cryopreservation of pollen will be applied to an increasing number of crop species, accompanied by the emergence of novel preservation technologies.

In addition, the thawing method is also an important factor in pollen viability (He et al., 2017; Jia et al., 2022). The thawing methods included thawing at room temperature, in a water bath (35°C), and under running water. A comparison of these methods showed that thawing under running water is superior to thawing under water bath, and thawing under water bath is better than thawed at room temperature (Jia et al., 2022). Thus, an improved thawing method is particularly important for enhancing pollen viability.

Ideally, pepper seed production can be completed in various sowing windows and in different areas for most of the year. Therefore, a sufficient supply of pepper pollen is a key factor for efficient seed production. However, current research on the long-term preservation of chili pollen while maintaining high viability has yet to provide a comprehensive solution. Bezdíčková (1989) showed that assessing the viability of stored pollen after 17 days in liquid nitrogen (-196°C) resulted in a decrease in pollen viability of about 50%, with no further significant changes in viability observed after storage for up to 82 days. Although early studies have demonstrated that cryogenic preservation is an effective method for the long-term storage of chili pollen, current technologies still fail to meet the practical demands of seed production in terms of storage duration, convenience, operability, and cost.

Pollen preservation techniques have been studied in several crops, which is essential for both conservation and agricultural applications (Dinato et al., 2020). However, research on the effective method for long-term cryopreservation of pepper pollen remains notably limited. This study establishes a comprehensive protocol for the cryopreservation of pepper pollen, including steps for pollen collection, dispersal, drying, sealing, pre-cooling, freezing, thawing, and hybridization. Our study results provide a scientific foundation for the long-term ultra-low temperature storage of chili pepper pollen and its application in hybrid breeding.

## Materials and methods

### Plant materials

The experiments were conducted at the vegetable station of the Institute of Tropical Eco-agriculture, Yunnan Academy of Agricultural Sciences, Chuxiong, Yunnan, China (25°69’ N, 101°87’ E, 1120.90 m). The pepper species used in this study was Fresh Pepper, with the maternal line labeled as 7A and the paternal line as 7B-1. All the experiments were conducted in a greenhouse in this study.

### Pollen collection period for peppers

**Figure 1:**
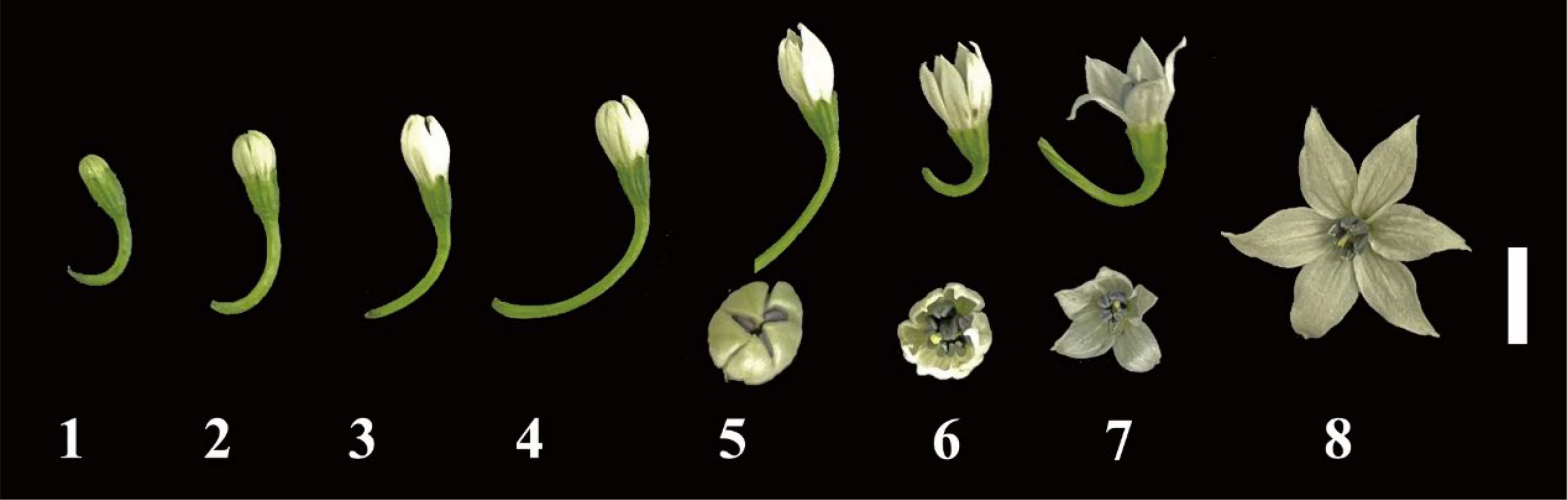
The development of pepper flowers. The white bar represents 1 cm.

Based on observations of pepper pollen development, we divided the process into eight stages, ranging from the bud phase to the flowering phase (**Figure 1**). The first stage was flower bud. Stages two to four represent the flower development stage, during which the flowers continue to grow, and both the flower buds and pollen develop simultaneously. The fifth stage indicates pollen maturation, during which the pollen was fully mature and exhibits high viability, although the flowers have not yet opened. Stages six to eight were the flowering stage, during which the flowers gradually open. By stage eight, the flowers reach full bloom, and all pollen grains from the anthers have dispersed. Ultimately, based on previous studies (Mathad et al., 2013; Jia et al., 2022) and our preliminary experiments, we identified stage five (unopened flowers) as the optimal time for pollen collection in this study.

### Collection of pepper pollen and dehydration treatment

(1) Pollen collection: The petals and sepals were removed, and the pistils were dried using a 100 W incandescent lamp for 30 minutes, with the lamp positioned 30–40 cm above the pistils (Temperature: 28°C), the pollen was separated out.
(2) Pollen drying treatment: The collected pollen was placed in a desiccator containing silica gel at 30°C for 6 hours until the water content of the pollen fell below 10%. The method for determining pollen water content (WC) is described as follows.
(3) Determination of Water Content (WC): Prior to the drying, the sample was weighed using an analytical balance, with the initial pollen weight recorded as FW. The pollen is then placed on sulfur paper and dried in a 30°C incubator. The weight (FW_i_) is measured at 2, 4 and 6 hours. The pollen water content (WC) was calculated using the following formula:
$$WC=\frac{FW-\text{FWi}}{\text{FW}}\;\times 100\%$$

### Pollen preservation in containers

In this study, 2 ml and 10 ml cryotubes were used as pollen storage. The detailed steps are shown below:

1. First, the dried pollen is wrapped in sulfur paper. Second, aluminum foil is wrapped the sulfur paper. Finally, a desiccant (silica gel) weighing 10–20% of the pollen weight is measured and placed together with the wrapped pollen into a 2 mL cryovial.
2. The inert gas (nitrogen) is used to displace the air within the pollen storage vial.
3. The 2 mL pollen storage vial is placed into a 10 mL cryovial, and a cryoprotectant solution (glycerol, 10%) was added to the 10 mL vial before sealing it.

### Ultra-low temperature storage

First, the sealed pollen storage containers were pre-cooled at 4°C for 2–4 hours. Second, the storage containers were frozen at -20°C for 4–8 hours. Next, the freezing continued at -40°C for 4–8 hours. Finally, the pollen was placed in an ultra-low temperature refrigerator at -80°C for long-term preservation.

### Thawing methods of pepper pollen

To ensure that the cryopreserved pepper pollen can withstand temperature fluctuations and maintain its viability, a stepwise thawing process was implemented. First, the frozen pollen was removed and allowed to thaw at -20°C for 10–12 hours. Subsequently, it was placed in an ice bath for an additional 4–6 hours. Finally, the pollen storage container was placed in running water for 30 minutes. After completing these steps, the pollen is ready for pollination operations.

### Hybridization experiment of pepper

The pepper pollen was collected in October 2022 and subjected to drying, dehydration, and pre-cooling treatments before being stored at -80°C for 1 year. In October 2023, the pollen was thawed and used for hybrid pollination. Simultaneously, the pollen collected from August to October 2023 was stored at 4°C and -20°C for 7, 14, and 60 days, respectively. Fresh pollen was used as a control, and pollination was synchronized. The hybrid pollination experiments were performed between 9:00 and 11:00 AM. For each treatment group, 20 pepper plants were selected, and 40 flowers per plant were pollinated. The amount of pollen applied to each flower was kept consistent, and all pollination procedures were performed by the same individual. All experiments were performed in three replicates. Calculation of the pollination rate:


$$PR=(\frac{Fn}{N})\;\times 100\%$$

PR represents the hybrid pollination rate, F_n_ represents the number of flowers that successfully developed into fruits after pollination, N represents the total number of flowers pollinated.

### Ultrastructural observation of pollen

We used a HITACHI floor-standing scanning electron microscope (FlexSEM1000) to observe structural changes in pollen preserved under different conditions. The integrity and morphology of the pollen wall were analyzed to evaluate the effects of ultra-low temperature preservation on pollen viability.

### Quality of hybridized seeds

To assess the quality of hybrid pepper seeds, we conducted statistical analyses of the 1000-seed weight, germination rate (GR), and germination index (GI) for seeds obtained from crosses using pollen subjected to different treatments. For each treatment, 100 seeds were randomly selected, with three replicates. Germination experiments were performed at a constant temperature of 25°C.


$$GR=(\frac{n}{N})\;\times 100\%$$

N represents the total number of seeds, n represents the number of germinated seeds.


GI=∑(Gt/Dt) 

G_t_ represents the number of seeds germinated on day t, and D_t_ represents the time.

### Statistical analysis

We performed calculations and statistical analyses by using R software, statistical significance was defined based on the Tukey HSD test with a threshold of *p* < 0.05.

## Results

### Ultrastructure of pepper pollen in different treatment conditions

**Figure 2:**
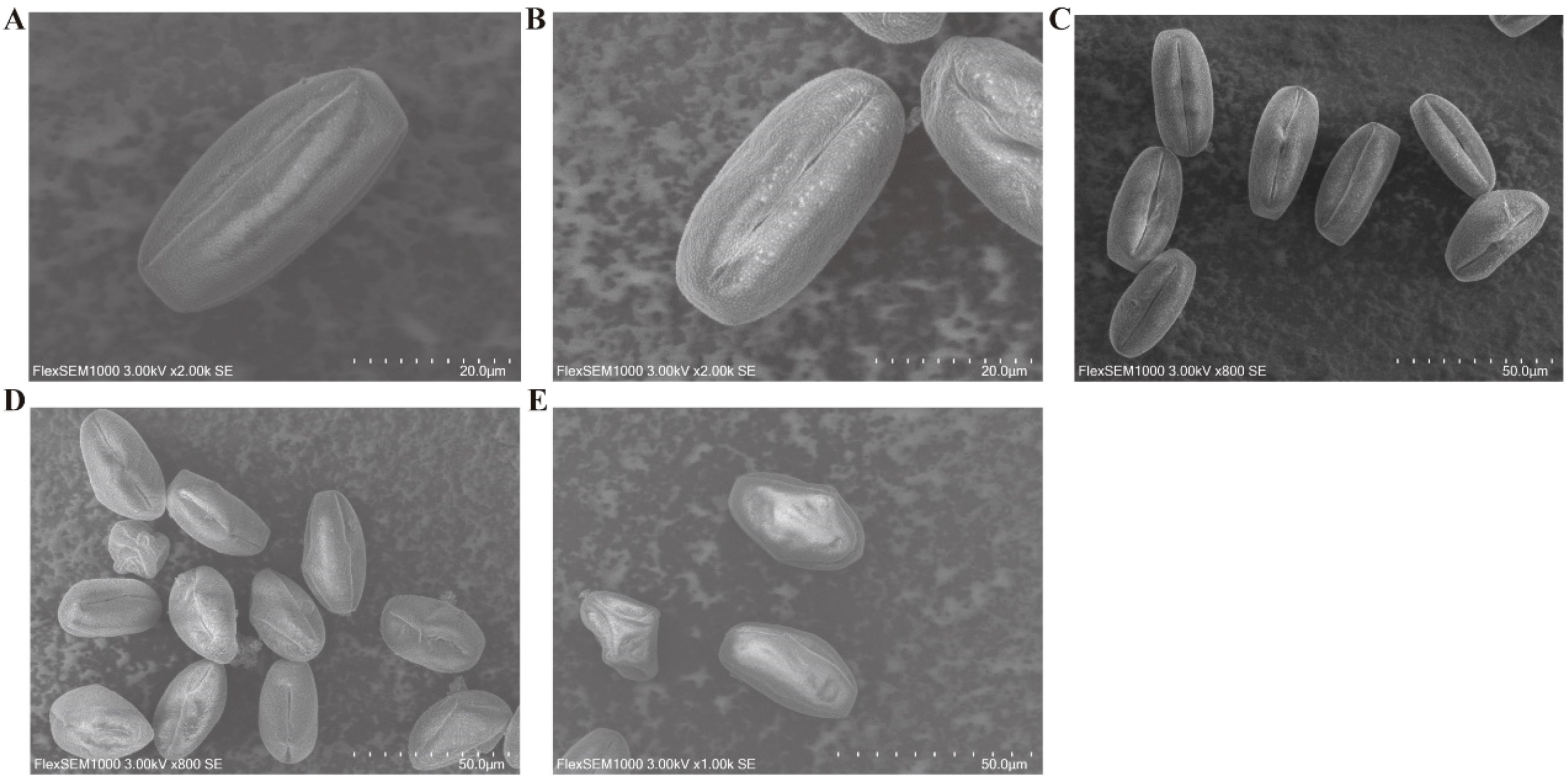
High-resolution ultrastructure of pepper pollen from different treatment groups. **(A)** Fresh pollen (CK). **(B)** -80°C/60d. **(C)** -80°C/1Y. **(D)** -20°C/60d. **(E)** 4°C/60d. Control (CK - freshly isolated pollen), pollen stored at -80°C for 60 days or one year (-80°C/60d, -80/1Y), and pollen stored at 4°C or -20°C for 60 days (-20/60d, 4/60d, respectively).

To visually observe the morphology and ultrastructure of pepper pollen under different storage conditions, we selected pollen samples from five treatment groups for scanning electron microscopy: CK, -80/1Y, -80/60d, -20/60d, and 4/60d. The results showed that the pollen stored at ultra-low temperatures (-80°C) retained a morphology like that of fresh pollen grains (**Figures 2A–C**), indicating that the pollen grains still possess high viability. However, the pollen stored at low temperatures (-20°C and 4°C) underwent significant structural changes, with most pollen grains shifting from a plump to a deflated state, suggesting a decline or loss of pollen viability (**Figures 2D, E**).

### Fruit development and ripening of pepper

**Figure 3:**
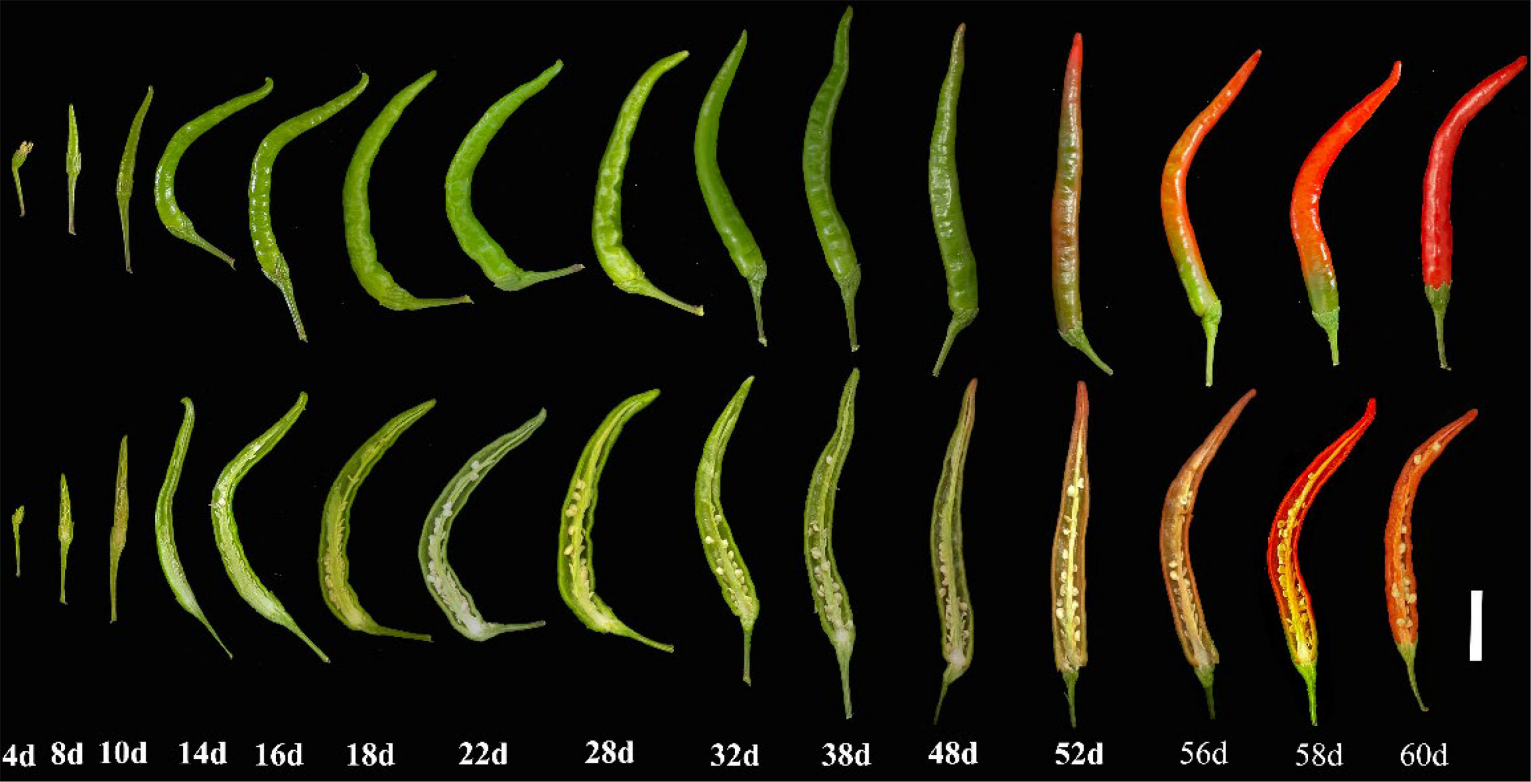
Fruit development and ripening period of hybrid peppers. The white bar represents 1 cm.

We systematically observed the development and ripening of hybrid pepper fruits (**Figure 3**). The growth cycle of pepper fruits is approximately 60 days, starting from the day of pollination. This cycle can be divided into two stages: the fruit development stage and the fruit ripening stage. The fruit development stage spans from 0 day to 38 day, during which the fruit rapidly enlarges, and the seeds progressively mature. The fruit ripening stage from 38 day to 60 day, during which the fruit size remains relatively stable, and the pericarp turned green to red. When the pericarp has fully turned red, the fruit can be harvested, and hybrid seeds can be collected.

### Effect of storage temperature and duration on pepper fertilization

**Figure 4:**
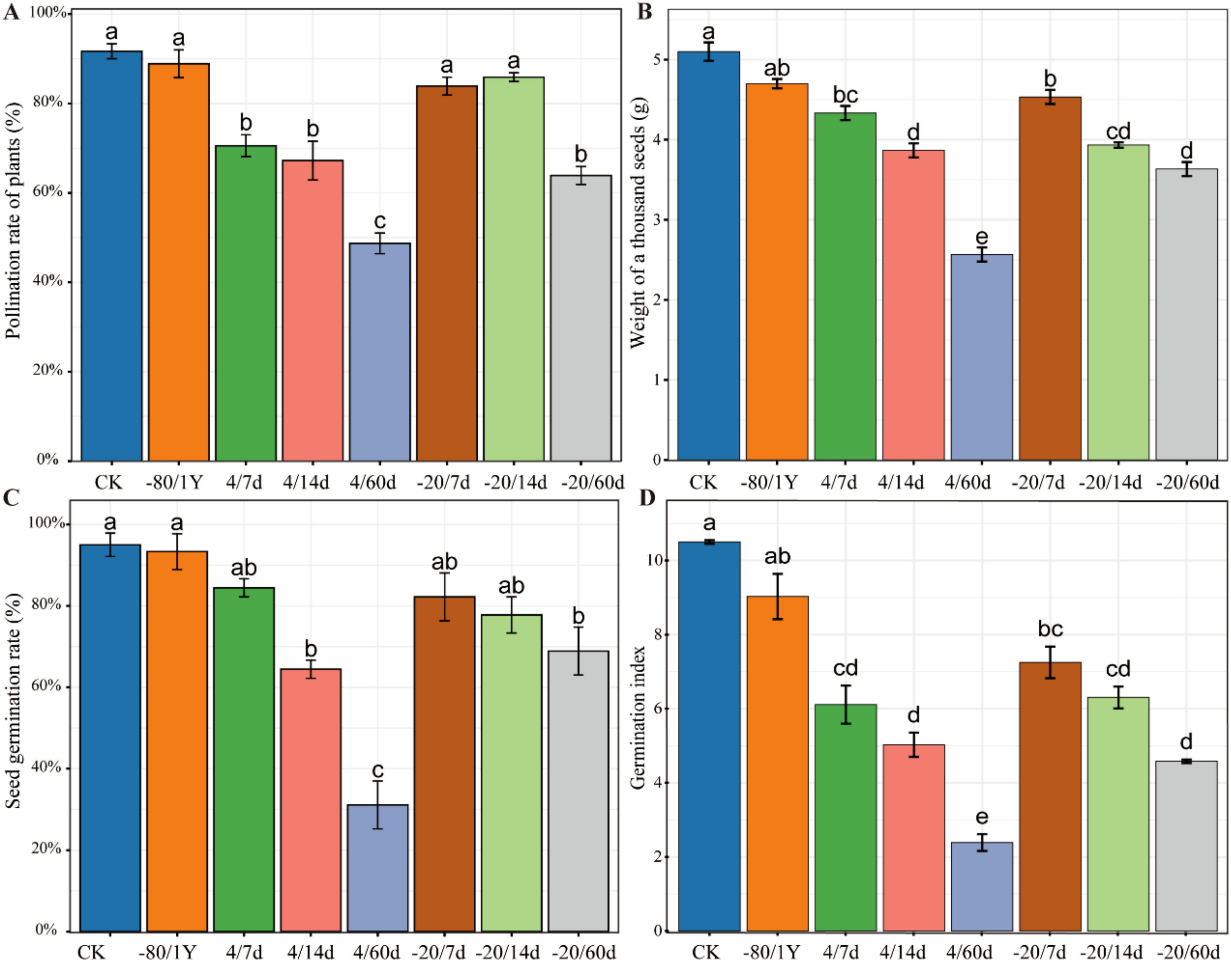
Fertilization success of pollen from different treatment groups and the quality of resulting hybrid seeds. **(A)** The pollination rate of plants. **(B)** The thousand-seed weight. **(C)** The germination rate of hybrid seeds. **(D)** The germination index of hybrid pepper seeds across different treatment groups. Control (CK - freshly isolated pollen), pollen stored at -80°C for one year (-80/1Y), and pollen stored at 4°C or -20°C for 7, 14 or 60 days (4/7d, 4/14d, 4/60d, -20/7d, -20/14d and -20/60d, respectively).

The quality of hybrid seeds is a key indicator for evaluating pollen viability. We conducted hybrid pollination using pollen from eight different treatments. The pollination rate was calculated based on the normal development of fruits following pollination. The results indicated that the pollination rate for CK and -80/1Y was more than 90%. Although the pollination rate of CK was higher than of -80/1Y, there was no significant difference (**Figure 4A**). Additionally, the pollination rates for the -20/7d and -20/14d groups were both above 80%. The pollination rates in the other treatment groups were low, indicating a significant loss of pollen viability.

In addition, we statistically analyzed the number of seeds per fruit of different treatment groups (**Supplementary Figure S1**, **Supplementary Figure S2**). The results indicated that there was no significant difference in the mean number of seeds per fruit between the CK and -80/1Y treatments. Specifically, the average seed numbers per fruit were 98 for the CK group and 93 for the -80/1Y group. Next, we compared the thousand-seed weight of hybrid seeds of eight treatments. The results showed that the CK had the highest values for thousand-seed wight, followed by -80/1Y (**Figure 4B**). And the comparison between the -20/7d and -80/1Y showed no significant difference in the thousand seed weight of hybrid seeds, indicating that pollen stored at -20°C for a short time retained high viability in pepper.

### Effect of ultra-low temperature pollen preservation methods on the germination rate of hybrid pepper seeds

**Figure 5:**
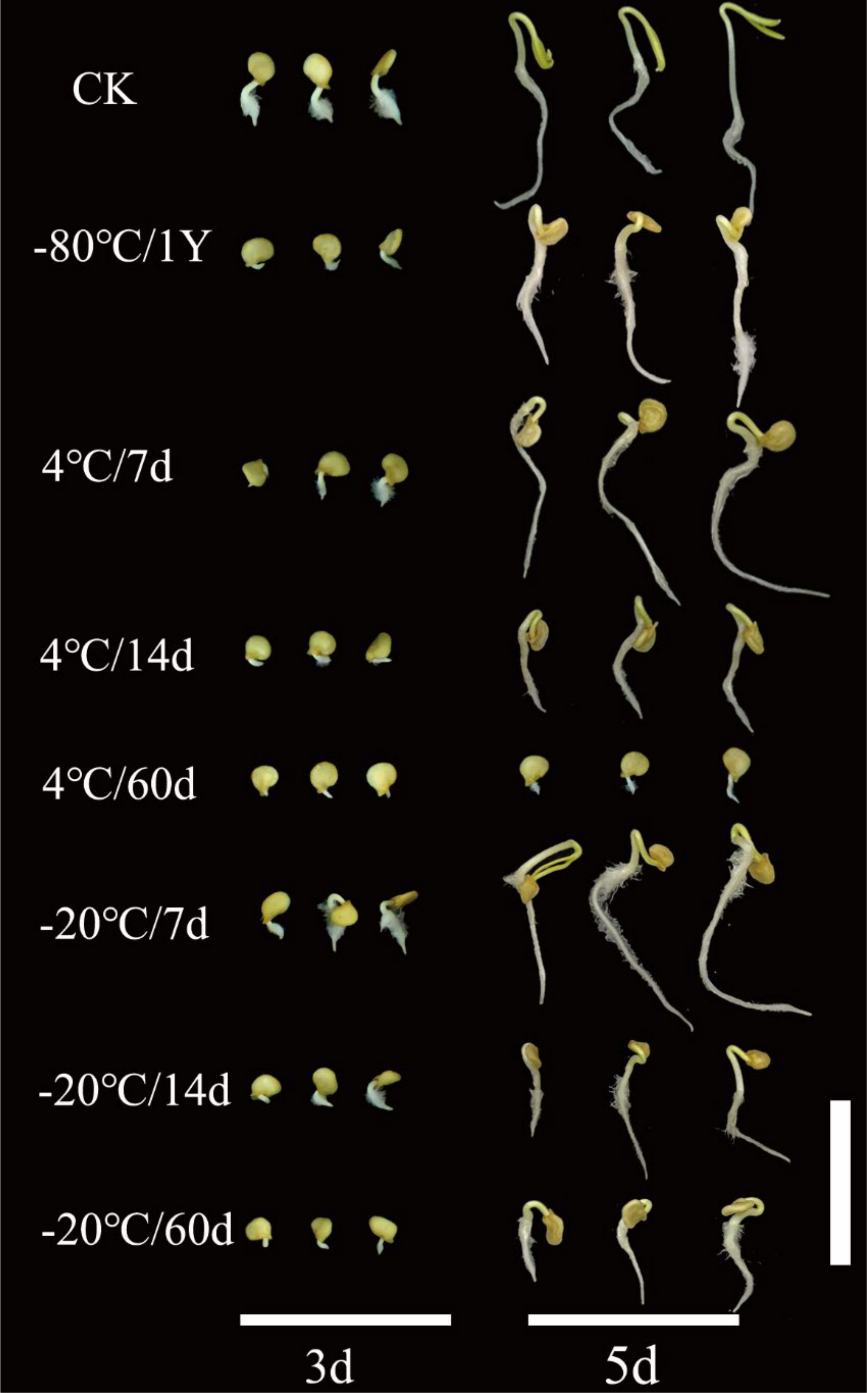
Germination of hybrid pepper seeds obtained by pollination with pollen from eight treatment groups at 3 and 5 days after sowing. The white bar represents 2 cm.

The results showed that the CK and -80/1Y had the highest GR and GI (**Figures 4C**, **D**). Additionally, the average GR for both the CK and -80/1Y was more than 90%, the average GR for the CK and -80/1Y was 95% and 93% respectively. Pollen stored at 4°C and -20°C for 7, 14, and 60 d can be used for successful pollination. However, the pollination rate of plants, thousand seed weight, GR and GI were significantly lower. Furthermore, during the entire germination period of the hybrid seeds, seedling development was poor in all treatment groups except for CK and -80/1Y (**Figure 5**). In conclusion, the pepper pollen preservation method used in this study effectively maintains long-term pollen viability.

## Discussion

Ultra-low temperature pollen preservation is a key tool in crop breeding and plant genetic resource conservation (Engelmann, 2004). This technique effectively maintains long-term genetic diversity and has demonstrated significant advancements in recent studies (Rajasekharan and Rohini, 2023a). As haploid carriers of paternal genetic information, pollen plays a vital role in crop breeding and improvement (Dinato et al., 2020; Rajasekharan and Rohini, 2023b). The technique is usually storage in liquid nitrogen (-196°C) (Engelmann, 2004; Nagar et al., 2023). However, due to the volatility of liquid nitrogen, it must be replenished periodically, which increases costs. Therefore, this study establishes a method for ultra-low temperature preservation of pollen that allows for the long-term maintenance of high viability in pepper pollen. This method is characterized by its simplicity, efficiency, and cost-effectiveness.

In addition, the process of pollen cryopreservation mainly includes pollen collection, dispersal, drying, freezing, and thawing (Jia et al., 2022). The selection of the period of pollen collection is also an important factor for ultra-low temperature preservation. Studies indicate that pollen collected from flowers that have not yet fully opened exhibits lower moisture content and a more stable cellular condition, making it more suitable for ultra-low temperature storage (Ganeshan et al., 2008; Karipidis and Douma, 2009; Jia et al., 2022). Thus, successful cryopreservation relies on effectively managing the water content of pollen (Fahy and Wowk, 2021; Faltus et al., 2024). In soybeans, a comparison of pollen collected from flowers at different stages (open and unopened) indicates that pollen collected from unopened flowers retains higher viability after long-term storage (Jia et al., 2022). Moreover, this has been confirmed in crops such as *Ziziphus moss* (Liang et al., 1993) and *Cornus florida* (Craddock et al., 2000). Additionally, Saha et al. (2023) showed that the pollen grains for cryopreservation in pepper should be collected from flower buds with a length of at least 4–6.5 mm to avoid contamination. Therefore, in our study, collecting pollen from unopened flowers proves to be both a reasonable and effective approach, as it enhances pollen viability and prevents pollen contamination (**Figure 1**).

The water content of pollen is also one of the key indicators for preservation, it is essential to use the correct method to control the water content of pollen (Dinato et al., 2020). During pollen cryopreservation, as the temperature decreases, free water inside pollen cells crystallizes into ice. If the water content is high in pollen, these ice crystals can damage the cellular structure, which affects both its quality and viability (Fonseca and Westgate, 2005; Liu and Wang, 2001; Shang et al., 2018). And it has been shown that dehydration at room temperature is more effective in maintaining pollen viability compared to dehydration under high-temperature conditions (D’antonio and Quiros, 1987; Rajasekharan and Ganeshan, 1994). However, the preservation of pollen is time-sensitive and pollen should be preserved as early as possible. Research indicates that when pollen is dehydrated using a constant-temperature oven, its moisture content can decrease to 11.0% within 10 hours, while preserving its optimal viability (Jia et al., 2022).Therefore, in this study, the water content of pepper pollen was maintained at less than 10% using constant-temperature oven. And we also placed a desiccant (silica gel) in the pollen storage container.

In practical applications, common methods for pollen dehydration include desiccators, incandescent lamp drying, freeze-vacuum drying, and air drying at room temperature (Fayos et al., 2022; Gómez-Mena et al., 2022). The selection of a specific method depends on the pollen species and the experimental requirements. Each technique presents distinct advantages and limitations, influencing both the efficiency of dehydration and the preservation of the pollen viability (Midilli et al., 2000). For example, desiccators provide a controlled environment that minimizes contamination. In contrast, incandescent lamp drying, though faster, may expose pollen to excessive heat, which could reduce germination rates (Mallikarjuna et al., 2021). Comparatively, freeze-vacuum drying is often associated with higher operational costs due to the specialized equipment required to maintain low temperatures and create a vacuum environment (Connor and Towill, 1993; Zhang et al., 2019). Therefore, researchers must select the most appropriate method for pollen drying based on the specific requirements of their experiments.

Furthermore, the pre-cooling and thawing of pollen are critical steps that must be carefully managed to prevent abrupt temperature fluctuations. Rapid cooling may increase the risk of ice crystal formation, whereas slow warming helps reduce osmotic shock (Matsumoto, 2017; Benelli, 2021). Therefore, in our study, before freezing, a stepwise cooling process was applied, as well as a stepwise warming process during thawing, to prevent pollen cells from being damaged by rapid temperature changes. In our study, the pre-cooling treatment includes three steps: 4°C (2–4h) → -20°C (4–8h) → -40°C (4–8h) →-80°C. During the thawing process, pollen should not be exposed to low-temperature conditions for extended periods, as free water may re-crystallize, leading to cell damage and a decline in pollen viability (Zhang et al., 2006; Dinato et al., 2020; Jia et al., 2022). In our work, the thawing treatment consists of two steps: -20°C (10–12 h) → ice water bath treatment (4–6 h). This approach has also been demonstrated in studies on the preservation of soybean and potato pollen (Zhang et al., 2006; Faltus et al., 2024). Moreover, compared to previous studies (Luna et al., 2001; Jia et al., 2022; Ren et al., 2019), our study incorporated inert gas (nitrogen) to replace the air in the storage container, thereby further reducing pollen respiration. Additionally, 10% glycerol was used as a cryoprotectant solution to prevent rapid temperature fluctuations in the storage container during fast freezing or thawing.

Previous studies have shown that pepper pollen loses its viability after 3 days of storage at room temperature. At -20°C, pollen can be stored for 6 days, while storage in liquid nitrogen (-196°C) allows pollen to be maintained for 47 days (Mathad et al., 2013). These results were obtained through pollen germination tests, a simple and rapid method. However, it is susceptible to human error and does not provide a clear understanding of fruit development following pollen fertilization. Hybrid pollination is the most effective method for assessing pollen viability, with its ultimate value reflected in the resulting hybrid seeds. In our study, pollen preserved for one year under ultra-low temperature conditions was used for hybrid pollination, with fresh pollen as the control. To ensure the completeness and comparability of the experiment, additional pollen treatment groups were included, and the pollination experiments were conducted simultaneously. The research results indicate that there is no significant difference in pollination rate and hybrid seed quality between -80/1Y and CK. In addition, by using the ultra-low temperature methods for pollen preservation, and pollination is not limited by time or location. For short-distance transport, pollen can be stored in ice boxes, while for long-distance transport, it can be shipped with dry ice. For long distances, pollen can be transported by dry ice.

In summary, while our developed ultra-low temperature preservation method for pepper pollen is more complex compared to traditional methods, the results in terms of pollen viability are highly satisfactory. Studies indicate that the effectiveness of cryoprotectants is related to their ability to penetrate cellular membranes (Dinato et al., 2020), such as dimethyl sulfoxide (Me_2_SO), glycerol, and propylene glycol, etc. Cryoprotectants play a crucial role in protecting pollen cells from damage during freezing and thawing processes. The application of cryoprotectants in plant cryopreservation is well-established. Kulus and Zalewska (2014) provided a detailed description of cryopreservation techniques applied to plants, including *Colocasia esculenta* (Thinh and Takagi, 2002), *Lilium* sp (Bouman et al., 2003), and *Saintpaulia ionantha* (Moges et al., 2004). Therefore, the development of a method that combines inert gases (such as nitrogen) with glycerol for ultra-low temperature pollen preservation is of great significance for advancing research on long-term pollen storage.

## Conclusion

In this study, we established a method for the ultra-low temperature preservation of pepper pollen, which is characterized by simplicity, convenience and efficiency. The results showed that pepper pollen was stored at -80°C for one year retains high viability. Compared with fresh pollen, there was no significant difference in pollination rate and hybrid seed quality. This technique not only extends the storage period of pollen but also ensures the preservation of its fertilization potential over extended periods. Thus, our study provides technical support for the long-term preservation of pepper pollen.

## Data Availability

The original contributions presented in the study are included in the article/**Supplementary Material**. Further inquiries can be directed to the corresponding authors.
