# Supplementary material for: Effect of ultra-low temperature storage on the viability of pepper pollen and its implications for hybrid breeding
Source: Front Plant Sci. 2025 Mar 26;16:1516016. doi: 10.3389/fpls.2025.1516016 (PMC12010900; doi:10.3389/fpls.2025.1516016)
Supplement: Supplementary file 1 [file SupplementaryFile1.pdf]

Supplementary Material

Effect of Ultra-low Temperature Storage on the Viability of Pepper Pollen and Its Implications for Hybrid Breeding

Kanghua Du [1†], Da Zhang [1†], Jixian Ma [1†], Zhong Dan [1], Xianqin Wen [3], Weiwu Lv [1], Long Yang [1], Lingfeng Bao [1], Yirong Li [1], Guangping Chen [1], Jie Zhang [2*] and Wanfu Mu [1*]

[1] *Institute of Tropical Eco-agriculture Yunnan Academy of Agricultural Sciences, Chuxiong, Yunan, 651300, China.*

[2] *College of Horticulture and Landscape, Yunnan Agricultural University, Kunming, Yunnan, 650201, China.*

[3] *Yunnan Seed Management Station, Kunming, Yunnan, 650031.*

[*] Corresponding authors: zhangjie@ynau.edu.cn (Jie Zhang); sinongmwf@126.com (Wanfu Mu).

[†] These authors contribute equally to this work.

Supplementary Figure Captions

Supplementary Figure S1. Hybrid pepper seeds under different treatment groups. The white bar represents 1 cm.

Supplementary Figure S2. Number of seeds per fruit of hybrid pepper under different treatment groups.

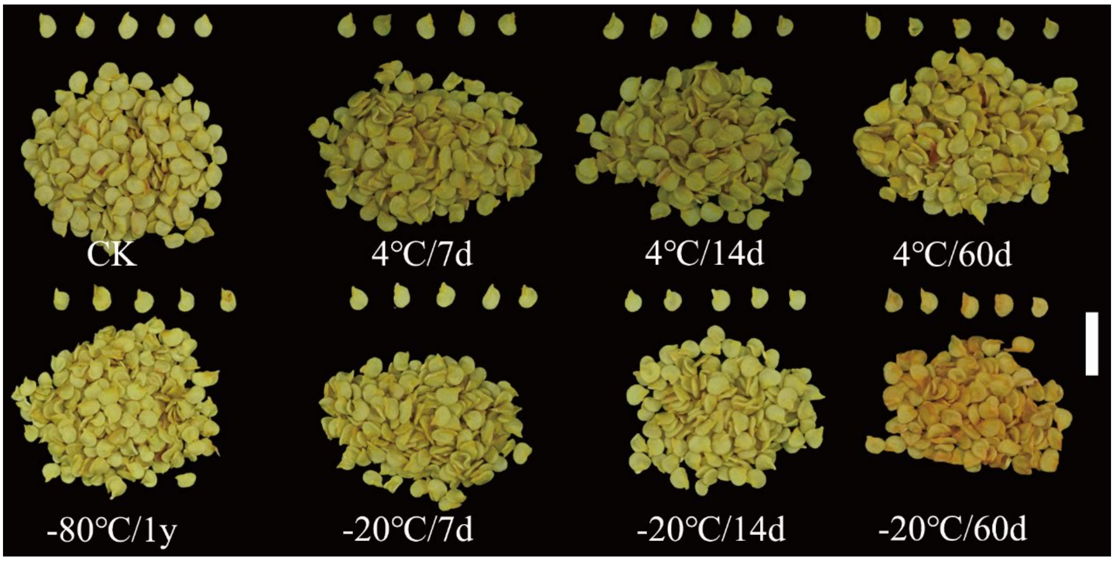

Supplementary Figures S1

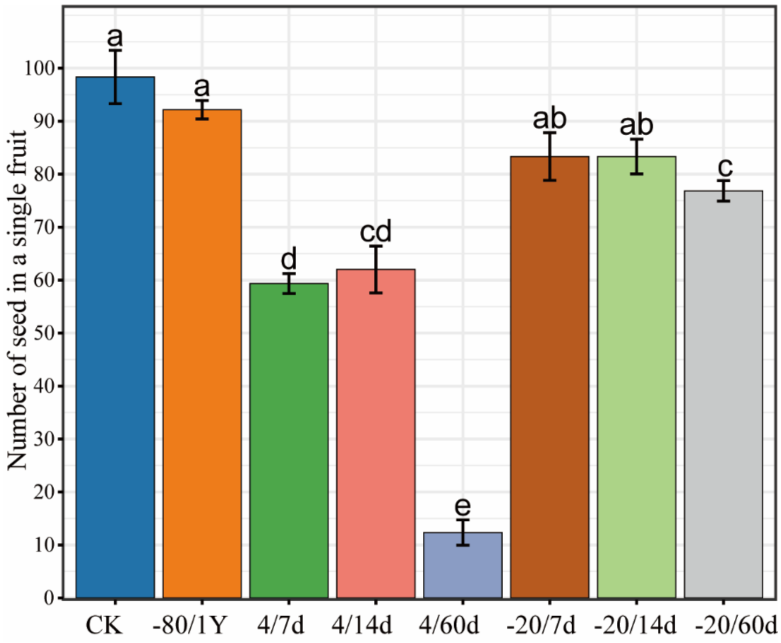

Supplementary Figures S2
